# De Novo Variants Found in Three Distinct Schizophrenia Populations Hit a Common Core Gene Network Related to Microtubule and Actin Cytoskeleton Gene Ontology Classes

**DOI:** 10.3390/life14020244

**Published:** 2024-02-09

**Authors:** Yann Loe-Mie, Christine Plançon, Caroline Dubertret, Takeo Yoshikawa, Binnaz Yalcin, Stephan C. Collins, Anne Boland, Jean-François Deleuze, Philip Gorwood, Dalila Benmessaoud, Michel Simonneau, Aude-Marie Lepagnol-Bestel

**Affiliations:** 1 Université Paris Cité, Institute of Psychiatry and Neuroscience of Paris (IPNP), INSERM U1266, 75014 Paris, France; yann.loemie@pasteur.fr (Y.L.-M.); caroline.dubertret@aphp.fr (C.D.); p.gorwood@ghu-paris.fr (P.G.); aude-marie.lepagnol-bestel@univ-reims.fr (A.-M.L.-B.); 2 Université Paris-Saclay, CEA, Centre National de Recherche en Génomique Humaine (CNRGH), 91057 Evry, France; plancon@cngrh.fr (C.P.); boland@cnrgh.fr (A.B.); deleuze@cnrgh.fr (J.-F.D.); 3 AP-HP, Department of Psychiatry, Louis Mourier Hospital, 92700 Colombes, France; 4 Laboratory for Molecular Psychiatry, RIKEN Center for Brain Science, Saitama 351-0106, Japan; takeo.yoshikawa@riken.jp; 5 Université de Bourgogne, INSERM Research Center U1231, 21000 Dijon, France; binnaz.yalcin@inserm.fr (B.Y.); stephan.collins@u-bourgogne.fr (S.C.C.); 6 GHU-Paris Psychiatrie et Neurosciences, Hôpital Sainte Anne, 75014 Paris, France; 7 Etablissement Hospitalo-Universitaire Spécialisé Psychiatrie Frantz FANON, Université Saad DAHLAB, Blida 09000, Algeria; benmessaoud_dalila@univ-blida.dz; 8 Laboratoire LuMin, FRE 2036, Universite Paris-Saclay, CNRS, ENS Paris Saclay 4 Avenue des Sciences, 91190 Gif-sur-Yvette, France; 9 Department of Biology, Ecole Normale Supérieure de Paris-Saclay, Université Paris-Saclay, 4 Avenue des Sciences, 91190 Gif-sur-Yvette, France

**Keywords:** schizophrenia trios, cohorts, mutation, gene ontology, cytoskeleton, microtubule, *Coro1c* gene, *Coro1c* mouse

## Abstract

Schizophrenia (SZ) is a heterogeneous and debilitating psychiatric disorder with a strong genetic component. To elucidate functional networks perturbed in schizophrenia, we analysed a large dataset of whole-genome studies that identified SNVs, CNVs, and a multi-stage schizophrenia genome-wide association study. Our analysis identified three subclusters that are interrelated and with small overlaps: GO:0007017~Microtubule-Based Process, GO:00015629~Actin Cytoskeleton, and GO:0007268~SynapticTransmission. We next analysed three distinct trio cohorts of 75 SZ Algerian, 45 SZ French, and 61 SZ Japanese patients. We performed Illumina HiSeq whole-exome sequencing and identified de novo mutations using a Bayesian approach. We validated 88 de novo mutations by Sanger sequencing: 35 in French, 21 in Algerian, and 32 in Japanese SZ patients. These 88 de novo mutations exhibited an enrichment in genes encoding proteins related to GO:0051015~actin filament binding (*p* = 0.0011) using David, and enrichments in GO: 0003774~transport (*p* = 0.019) and GO:0003729~mRNA binding (*p* = 0.010) using Amigo. One of these de novo variant was found in *CORO1C* coding sequence. We studied Coro1c haploinsufficiency in a *Coro1c^+/−^* mouse and found defects in the corpus callosum. These results could motivate future studies of the mechanisms surrounding genes encoding proteins involved in transport and the cytoskeleton, with the goal of developing therapeutic intervention strategies for a subset of SZ cases.

## 1. Introduction

Schizophrenia (SZ) is a devastating mental illness characterized by positive symptoms (such as hallucinations and delusions), negative symptoms (such as social withdrawal, avolition, anhedonia, and self-neglect), and cognitive deficits (including impairments in executive function and attention) [1]. This heterogeneous and complex psychiatric disorder, characterized by severe cognition, emotion, and social functioning impairments [2], affects 1% of the global population. In addition to environmental factors, although the aetiology of schizophrenia remains elusive, it is known to be a strong genetic component. However, the genetic explanation of the majority of schizophrenia cases remains unresolved. Current theories emphasize the contribution of large numbers of common genetic variants of small effect, combined with rare variants of larger effect. Intensive research has revealed a number of candidate genes that may be involved in SZ; however, the degree of genetic association tends to be inconsistent.

Numerous genetic association studies have made great steps towards the identification of either common or rare variants associated with SZ [3,4,5,6]. Mutations that confer substantial risk for SZ have been identified at several loci, most of which have also been implicated in other neurodevelopmental disorders, including autism spectrum disorder [7]. Studies involving next-generation sequencing technology have provided preliminary evidence that de novo single-nucleotide mutations might also increase the risk of SZ. However, these are very small in scale [8]. Exome sequencing is a powerful tool for identifying mutations on specific genes, predicting consequences of such coding region mutations on gene function, and also predicting the effect of rare or de novo mutation on SZ risk [9]. Exome sequencing performed in patients with SZ indicated that a variety of distinct genes are influence the disease. For instance, 15 de novo mutations were identified in 14 patients [10], and 40 de novo mutations were identified in 27 patients, affecting 40 genes [11]. Furthermore, exome sequencing and whole-exome sequencing (WES) has been performed for 134 and 32 patients, respectively with the identification of 5155 variants [12]. These results indicate that SZ risk is unlikely to be predominantly influenced by variants just outside the range detectable by genome-wide association studies (GWASs). Rather, multiple rarer genetic variants must contribute substantially to the predisposition to SZ, suggesting that both very large sample sizes and gene-based association tests will be required for securely identifying genetic risk factors. However, with the exception of very rare variants, each shows a relatively small contribution to disease risk, and few of them were able to understand the biological effect. Thus, neuropathophysiology and biological mechanisms remain largely unknown. Our study supports the idea that the genetic architecture of neuropsychiatric disorders includes a constellation of rare mutations in many different genes. This “common disease—rare alleles” hypothesis [13] is also supported by findings in human genomics [14].

Here, we applied the next-generation deep sequencing of SZ by using family trios with the goal of identifying de novo mutations associated with SZ and novel rare disease-predisposing variants. Using bioinformatics analysis methods, we first predicted the most damaging de novo mutations, and second, we identified the gene network involved. Finally, we compared our biological pathway and gene network linked to SZ with those obtained performing a meta-analysis on published GWASs, copy number variation (CNV), and single nucleotide variant (SNV) mutations.

## 2. Materials and Methods

### 2.1. BioInformatics Analysis

To identify functional gene network involved in schizophrenia, we applied the NETwork-Based Analysis of Genomic variations (NETBAG+) computational method described in [15,16]. This approach enables the processing of diverse types of genetic variations. We included GWAS loci (as CNV events), genes carrying de novo mutations from recent study and recurrent CNVs highlighted in meta-analysis. We selected the best network according to the minimal adjusted *p*-value and maximal *z*-score. In order to characterize the NETBAG+ networks, we then performed functional analysis of the gene nodes. We used the functional annotation chart of the DAVID Bioinformatics resource with a default Human background. We used the BioGRID database [17] (with all interactions of the protein UBC removed) and cytoscape software 3.2.1 to represent our networks. We kept genes with GO annotations “microtubule-based process”, “actin cytoskeleton”, and “synaptic transmission” or “synapse” and their direct interactors as part of the network. We performed FATIGO analysis with the Babelomics 4 web server. We used a dataset of genes of three diseases (SZ, ID, and ASD) reported in a recent study [18]. We used all RefSeq genes mapped to the Human genome (hg19) as a background. We built the Venn diagram according to these analyses with the R package “VennDiagram”.

### 2.2. Clinical Samples and DNA Preparation

Clinical samples consisted of three family-based populations of 91 Algerian, 54 French, and 74 Japanese patients with SZ, with their biological parents. In total, 219 patients were evaluated by trained psychiatrists using the Diagnostic Interview for Genetic Study [19], a semi-structured interview that assesses the lifetime Diagnostic and Statistical Manual of Mental Disorders Volume IV criteria of schizophrenia and other psychiatric diseases. In addition, the Scale for the Assessment of Positive and Negative (SANS) Symptoms were used to characterize the predominant clinical symptoms of the patients [20,21]. Descriptions of the Algerian and French samples have been reported elsewhere [22,23], as well as their demographic and clinical characteristics [24]. This study was approved by the National Ethics Committees of Algeria, France, and Japan, and all individuals, probands and parents, gave written informed consent for their participation. Genomic DNA was extracted from peripheral blood samples from all probands and parents.

### 2.3. Whole-Exome Sequencing

We performed whole-exome sequencing (WES) using the Illumina HiSeq technique of next-generation sequencing. We create the exome-wide libraries from DNA using the SureSelect AllExome V4 50Mb from Agilent (Santa Clara, CA, USA). Then, we generated paired-end sequence data using Illumina HiSeq machines (Illumina, Inc., San Diego, CA, USA) (8plex sequencing).

### 2.4. De Novo Mutation Identification

De novo mutation identification was performed using a Bayesian approach. We first had to filter the false positives using a homemade method based on a Bayesian approach. We thus apply three successive steps: (i) definition of two classes using DNA chip and exome sequencing data; (ii) arrangement of the two classes according to their mean cover per site; and (iii) separation of the heterozygotes and homozygotes. These three steps filters applied, we built GATK parameter empiric distribution and gathered them.

### 2.5. Sanger Sequencing

Validation of de novo mutations was performed by Sanger sequencing. Sequence alignment and SNP detection were performed using Genalys software (GenalysWin2.8.3b) [25].

### 2.6. Polymorphism Phenotyping

We used PolyPhen-2, which predicts the possible impact of an amino acid substitution on the structure and function of a human protein using straightforward physical and comparative considerations. http://genetics.bwh.harvard.edu/pph2/ (accessed on 12 February 2023).

### 2.7. NETwork-Based Analysis of Genomic Variations

The NETBAG phenotype network finds relationships between genes by using a naïve Bayesian network. NETBAG scores the predicted likelihood that two human genes share the same phenotype. In doing so, NETBAG can uncover disease risk genes among a list of mutations observed in probands. The algorithm searches for cohesive clusters of genes perturbed by disease-associated genetic variations. Among a list of provided genes, NETBAG will search for a strongly interconnected subset of genes. Starting with each input gene as a seed node, a greedy search algorithm will choose the most strongly connected gene to add to the candidate set. Candidate networks are assigned a score based on a weighted sum of their edges, representing the likelihood that the respective genes participate in the same genetic phenotype. Network significance is then determined by comparing this score to a distribution of scores obtained by applying the same search algorithm to sets of random genes. http://innovation.columbia.edu/technologies/cu14362_network-based-analysis-of-genomic-variations (accessed on 12 February 2023).

### 2.8. Coro1c^+/−^ Mouse Brain Analysis

Mouse brains were removed following anaesthesia and decapitation. In all steps of these neuroanatomical studies, the animal’s genotypes were blinded to the experimenters. Standard operating procedures are described in more detail elsewhere [26]. Mouse brain samples were immersion-fixed in 10% buffered formalin for 48 h, before paraffin embedding and sectioning at 5 μm thickness using a sliding microtome (Leica RM 2145, Leica RM 2145; Leica Microsystems GmbH, Wetzlar, Germany).

Coronal sections were collected at Bregma +0.98 mm and −1.34 mm, according to the Allen Mouse Brain Atlas [27]. 

Brain sections were double-stained using luxol fast blue for myelin and cresyl violet for neurons, and scanned at cell-level resolution using the Nanozoomer whole-slide scanner (Hamamatsu Photonics, Shizuoka, Japan). Co-variates, such as sample processing dates and usernames, were collected at every step of the procedure and used to identify data drifts. Using in-house ImageJ plugins, an image analysis pipeline was used to standardize measurements of areas and lengths. Each image was quality-controlled for the accuracy of sectioning relative to the reference atlas and controlled for asymmetries and histological artefacts.

At Bregma +0.98 mm, the brain structures assessed were as follows: the total brain area; the lateral ventricles; the cingulate cortex; the genu of the corpus callosum; the caudate putamen; the anterior commissure; the piriform cortex; the primary motor cortex; and the secondary somatosensory cortex. At Bregma −1.34 mm, a maximum of 18 brain structures were assessed: the total brain area; the lateral and third ventricles; the retrosplenial granular cortex; the corpus callosum; the dorsal hippocampal commissure; the amygdala; the piriform cortex; the internal capsule; the optic tract; the mammillothalamic tract; the fimbria; the habenular nucleus; the hippocampus; the primary motor cortex; the secondary somatosensory cortex; the hypothalamus nucleus; the arcuate nucleus; and the 3rd ventricle ventral part.

All samples were also systematically assessed for cellular ectopia (misplaced neurons). Statistical comparison was performed using *t*-tests.

## 3. Results

### 3.1. Over-Representation of the Microtubule-Based Process Gene Ontology in the Schizophrenia NETBAG+ Cluster

To elucidate functional networks perturbed in SZ, we applied NETBAG+ to a set of genes affected by de novo SNVs (*n* = 609) [18] and CNVs (*n* = 58) [28]. Furthermore, we used GWAS data from a multi-stage SZ GWAS involving up to 36,989 cases and 113,075 controls, and were able to identify 346 genes from the 108 loci found associated with SZ [29] (Supplementary Table S1). All the mutations or loci associations used as input for our analyses were obtained from a dataset generated by genome-wide methodologies in order to induce no bias linked to pre-existing hypothesis. 

Types of variants (SNV, CNV, or GWAS) are reported in bracket after each gene. Interestingly, the Synaptic Transmission GO category is over-represented in the SZ GWAS repertoire (6 genes out of 24 in the list; *p* = 0.021), which is not the case for the MBP GO and Actin Cytoskeleton GO categories (1/25 and 1/23, respectively).

**Figure 1:**
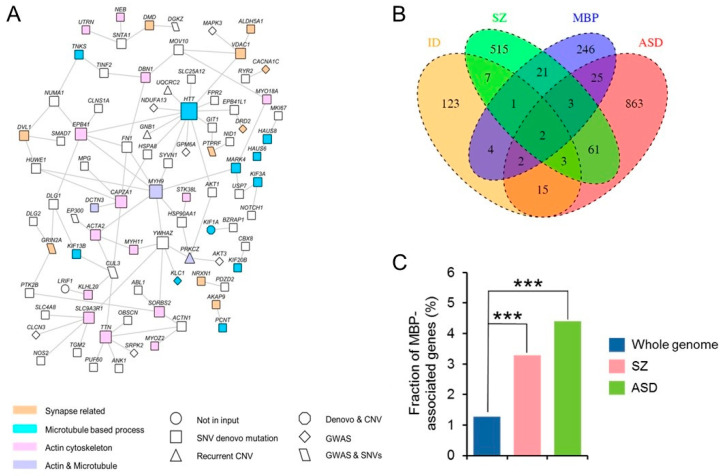
Enrichment of Gene Ontology GO:0007017; “Microtubule-Based Process” category in schizophrenia (SZ) and in autism spectrum disorder (ASD). (**A**) The network implicated by NETBAG+ based on SZ-associated 613 SNV, 63 CNV, and 360 GWAS from 108 loci identified a principal network (network comprises 559 genes, *p* = 0.012) displaying (i) genes belonging to the three highest-connected GO classes: Microtubule-Based Process (blue), Actin Cytoskeleton (pink), and Synaptic Transmission (orange); and (ii) direct interacting genes (white) from BioGRID direct protein–protein interactions (PPIs). Diamond symbols represent genes from GWASs; triangles represent genes from CNVs; rectangles are associated with genes from SNVs; octagons indicate genes from both SNVs and CNVs; and parallelograms pertain to genes from both GWAS and SNV mutation types. (**B**) Overlap of genes bearing de novo mutations in SZ, ASD, and intellectual disability (ID) disorders with genes of the GO:0007017; “Microtubule-Based Process” category. The overlap area in the Venn diagram shows the number of genes between/among different disorders and MBP (**C**) Bar plot showing the enrichment of MBP in SZ and ASD (SZ: *p* = 0.00013 and ASD: *p* = 0.00049). *** *p* < 0.001.

From this 1014-gene repertoire, we found that the largest network comprised 559 genes (*p* = 0.012) (Figure 1). We used DAVID17 to identify Gene ontology (GO) terms that were significantly enriched among network gene annotations (Table 1 and Table 2). This analysis identified three subclusters that are inter-related and with small overlaps, including the Microtubule-Based Process (MBP), Actin Cytoskeleton, and Synaptic Transmission. The microtubule subcluster (GO: 0007017; MBP; Table 2) contained genes that are associated with trafficking, such as MARK4, which phosphorylate microtubule-associated proteins, KIF1A, KIF13B, or KIF20B, which are members of the kinesin family; DNAH1, DNAH3, and DNAH9 which are members of the dynein family; and DCTN3, another member of the DCTN protein family essential for dynein activity in vivo. Here, 7 KIF genes were part of the 559-gene network, indicating a statistically significant enrichment (*p* < 0.0001) for the KIF superfamily that included 45 genes (http://www.genenames.org/genefamilies/KIF accessed on 12 February 2023). The actin subcluster (GO: 00015629; Actin Cytoskeleton) contained actin-related proteins (ACTA2, CAPZA1, CTNNA2) and myosins (MYO15A, MYO18A or MYO18B). The synapse subcluster (GO: 0007268; Synaptic Transmission) contained synaptic adhesion molecules such as neurexin (NRXN1); components of the presynapse (RIMS1); receptors of glutamatergic synapses (GRM3, GRIN2A, and GRID2); AKAP9, that directly interacts with NMDA receptors; and voltage-gated calcium channels (CACNA1C and CACNB2). It also included the dopaminergic receptor (DRD2) that is the target of all effective antipsychotic drugs. Interestingly, the GO Synaptic Transmission category showed an over-contribution (6 over 24; *p* = 0.021) of GWAS genes, namely, GRM3, GRIN2A, CACNA1C, CACNB2, PTPRF, and DRD2, whereas GWAS genes only contributed to 1/25 and 1/23 for the GO MBP and GO Actin Cytoskeleton categories, respectively (Table 2). Therefore, to explore the respective contribution of common variants versus rare variants, we separately analysed the SZ GWAS set and SZ SNVs + CNVs set. For SZ GWAS genes, we only obtained an over-representation of GO: 004274 A presynaptic membrane enrichment category (*padj* = 0.03) using Babelomics v4.2 19 and no significant GO category were obtained using DAVID. 

**Figure 2:**
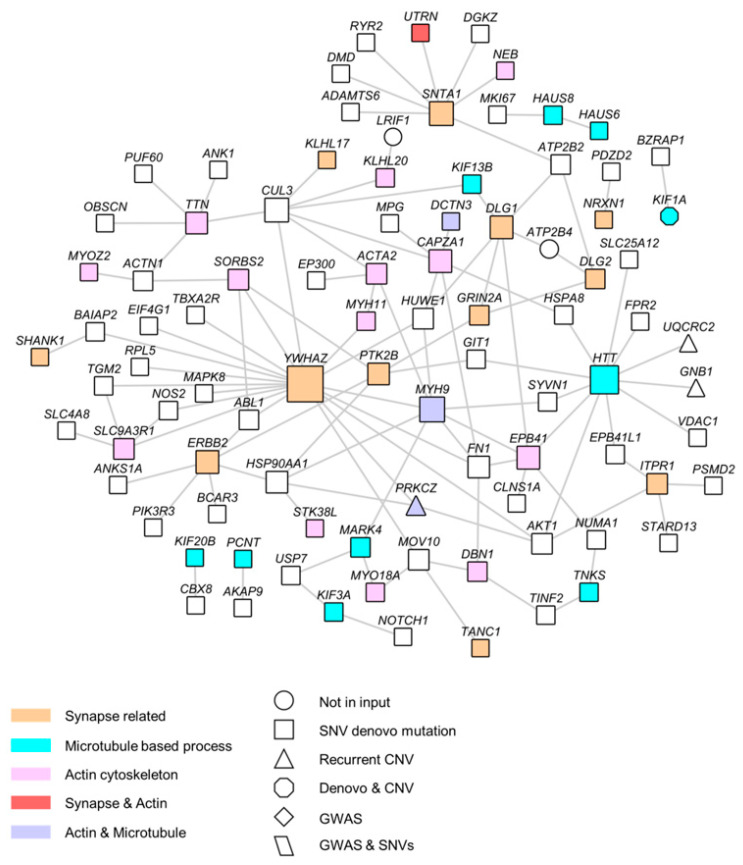
Full network of 292 genes based on the largest network from NETBAG+, which comprised 559 genes (*p* = 1.2 × 10^−2^).

We then performed NETBAG+ searches using only the set of 702 genes that included de novo SNVs (*n* = 609) and CNVs (*n* = 58). For this repertoire, the largest network comprised 572 genes (*p* = 0.0028) (Figure 1 and Figure 2, Supplementary Figure S1). The analysis identified three similar subclusters: MBP, Actin Cytoskeleton, and Synapse Part. Altogether, these results provide evidence that the different sources of genetic variations reinforce each other, as previously reported in [16].

### 3.2. WES from Three Different Ethnical Cohorts and De Novo Variant Identification

**Figure 3:**
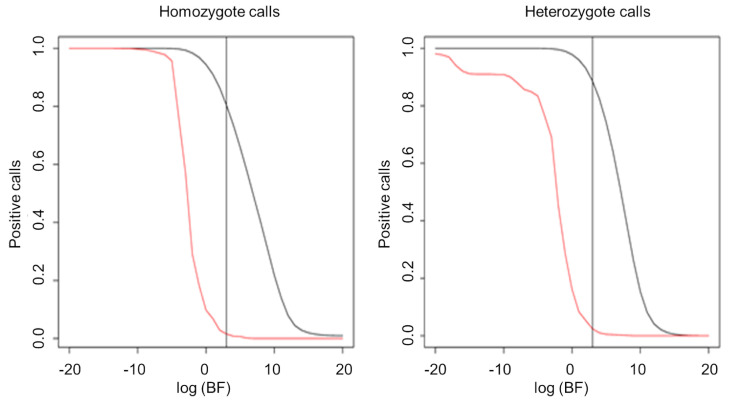
Bayesian factor. Proportions of validated mutations according to score (BF). The curves were computed on sites where we had both sequencing information and microarray calls. In red, discordant site (i.e., not the same call on two technologies, “false positives”); in black, concordant sites (“true positives”). We fixed the parameter in order to have ~5% of false positives.

We performed WES using Illumina technology in three distinct SZ cohorts: 91 Algerian, 54 French, and 74 Japanese SZ patients, with their two parents leading to a total of 657 sequenced DNA samples. All the DNA samples were controlled for quality and quantity before being loaded on sequencers. After sequence alignment and calling, performed with the GATK suite, only 75 Algerian, 45 French, and 61 Japanese trio families could finally be analysed and used for de novo variant identification (Table 3). De novo mutation identification was performed using a Bayesian approach. We estimated the Bayer factor (BF) for each single sequence (Figure 3) that enabled us to apply a new filter step on the BF: log(BF) < 3 and death-reading < 20.

From the 181 SZ trios, we identified 386 de novo mutations. We then validated 88 by Sanger sequencing: 35 in French, 21 in Algerian, and 32 in Japanese SZ patients (Table 3). These 88 de novo mutations were identified in 72 SZ patients (30 French, 17 Algerian, and 25 Japanese) localized on 22 different chromosomes, and only chromosomes carrying no de novo mutations.

We then looked into the distribution in percentages of the de novo mutations according to (i) the number of patients (Table 4) and (ii) the number of chromosomes (Table 5) carrying de novo mutation(s). While no difference was observed for the first distribution with almost 40% of patients, whatever the cohort, i.e. carrying mutation(s) or only one for the majority of them, a slight difference was shown for the second distribution. There was still the same trend for the total percentage of chromosomes carrying mutation(s) (61%, 70%, and 78% in the French, Japanese, and Algerian cohorts, respectively), with the majority of chromosomes carrying only one de novo mutation; however, there was a contrasting distribution for the percentage of chromosomes carrying several mutations. In fact, whereas the French cohort showed 17% with two mutations and only 4% with three or more, the two other cohorts, Algerian and Japanese, both displayed 4% with two mutations, and 22% and 26%, respectively, with three or more.

From these results, we decided to combine all de novo mutations from the three cohorts and analysed them together.

### 3.3. SZ De Novo Variant Analysis: Damaging Impact Prediction and Gene Network Identification

**Figure 4:**
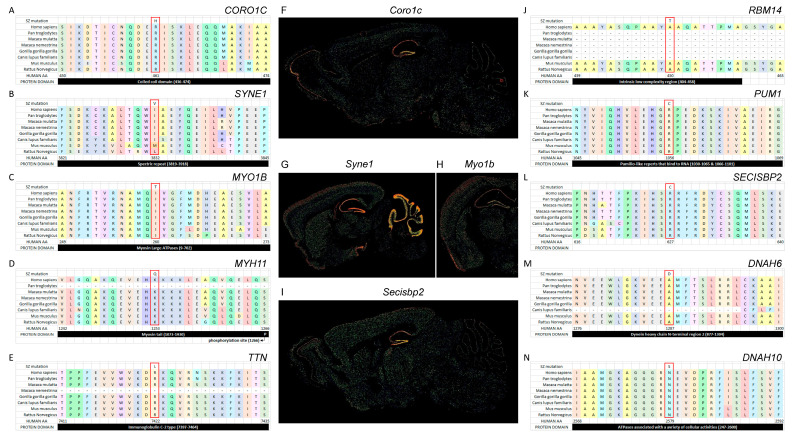
Multiple protein sequence alignment from rodents to primates indicating impact on conserved protein domains linked to de novo mutations. We show alignment for ten proteins—CORO1C, SYNE1, MYO1B, MYH11, TTN, RBM14, PUM1, SECIPBP2, DNAH6, and DNAH10—with the impact of the mutations in conserved protein domains (**A**–**E**,**J**–**N**). Alignments were performed using Clustal suite. Note the localized expression of CORO1C, MYO1B mouse orthologs in hippocampus, the brain region involved in memory and cognition (data from mouse Allen brain atlas) (**F**–**I**).

**Figure 5:**
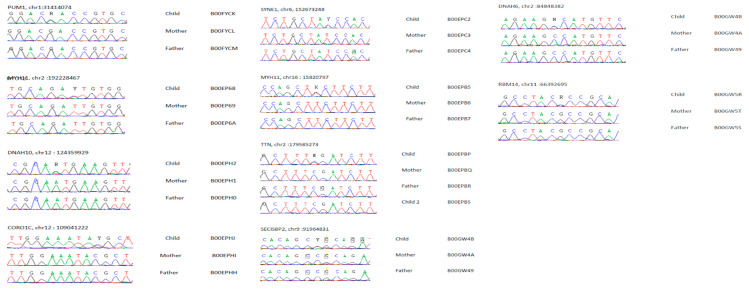
Sanger sequencing of regions surrounding de novo mutations in studied SZ trios. Presented here are the Sanger sequencing results for ten genes encoding CORO1C, SYNE1, MYO1B, MYH11, TTN, RBM14, PUM1, SECIPBP2, DNAH6, and DNAH10. Sequence alignment and SNP detection were performed using Genalys software (GenalysWin2.8.3b) [25].

We ran two complementary bioinformatics analyses to characterize our de novo SZ mutations. First, we used the polymorphism phenotyping PolyPhen-2 (PP2) software tool (http://genetics.bwh.harvard.edu/pph2/) to predict the possible impact of an amino acid substitution on the structure and function of a human protein. We were then able to rank the de novo variants according to the PP2 score, establishing three classes of mutations: (i) probably damaging, (ii) possibly damaging, and (iii) benign. Considering the three cohorts as distinct populations or taken together (Table 6), the majority of the de novo mutations were ranked as probably damaging, except for the Japanese cohort (only 37% of the de novo variants were ranked probably damaging). The entire list of de novo variants with their PP2 score is given (Table 7). Second, we used GO tools (David and Amigo/Panther) (Supplementary Table S2) to identify enrichment in a given GO class. For the Algerian cohort, we evidenced an enrichment in genes encoding proteins related to GO:0051015~actin filament binding (*p* = 0.0011) using David. For the sum of the three cohorts, we detected an enrichment for the same class GO:0051015~actin filament binding (*p* = 0.0020) using David. Panther analysis identified an enrichment in G0:0003774~transport (*p* = 0.019) and GO:0003729~mRNA binding (*p* = 0.010). We thus identified gene networks based on our de novo variants for classes similar to the biological pathways identified in the meta-analysis (Figure 1 and Figure 2, Supplementary Figure S1). Mutations of de novo variants are identified in conserved domain regions of the proteins, as illustrated by alignments of proteins from rodents to primates. We show this alignment for ten proteins: CORO1C, SYNE1, MYO1B, MYH11, TTN, RBM14, PUM1, SECIPBP2, DNAH6, and DNAH10 (Figure 4A–E,J–N). Note the localized expression of CORO1C, MYO1B mouse orthologs in hippocampus, the brain region involved in memory and cognition (Figure 4F–I). Furthermore, we validated these 10 de novo variants for each trio (mother, father, and patient) (Figure 5).

### 3.4. Functional Analysis of Mouse Coro1c Haploinsufficiency in the Brain: Coro1c Tm1a Mice Show Mild Neuroanatomical Defects Pertaining to the Corpus Callosum

We identified a possibly damaging *CORO1C* mutation in one patient of our cohorts (Figure 4A). The *CORO1C* gene is part of GO:0051015~actin filament binding category and encodes a WD40-repeat (WDR) protein [30].

**Figure 6:**
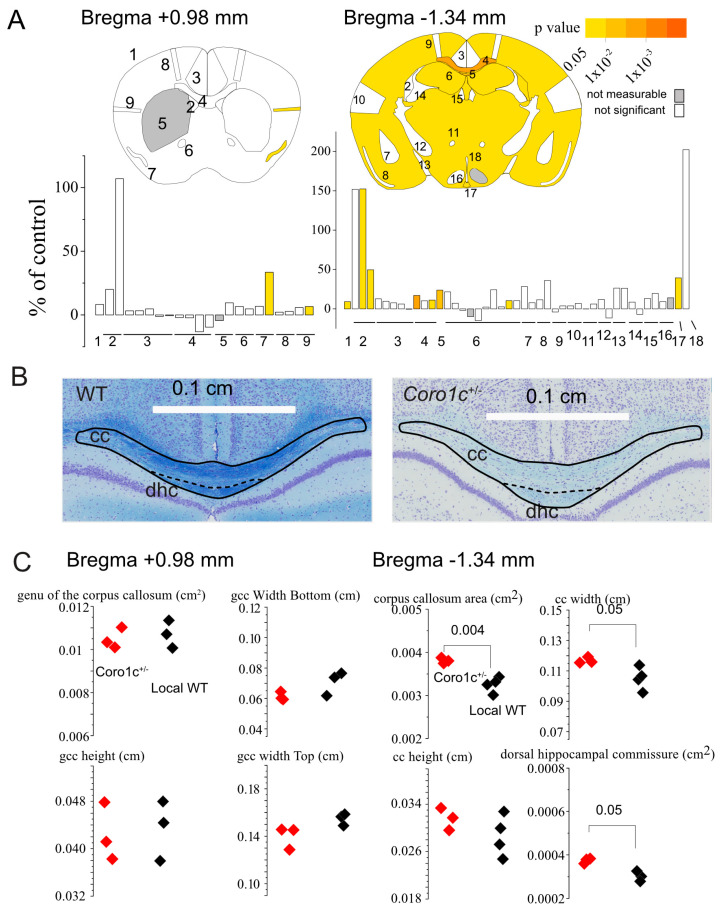
Coro1c tm1a mice show mild neuroanatomical defects pertaining to the corpus callosum. (**A**) Coronal planes at Br. +0.98 and −1.34 mm with numbered measurements shown on histograms are expressed as percentages from wild-type brain corresponding structures. Positive and negative values correspond to increased and decreased measurements relative to WT, respectively. The colour code indicates the significance threshold: white when not significant; grey when not computable. A list of neuroanatomical parameters and corresponding numbers is provided as the X-axis (a full description of the parameters is also provided in Supplementary Table S3). (**B**) Nissl-stained coronal brain sections from a *Coro1c^tm1a^* mouse (right) against WT (left). Corresponding scale bars are shown on each panel. (**C**) Box plots focusing on *corpus callosum* measurements at Br. +0.98 and −1.34 mm. Statistics indicated are *t*-tests.

Neuroanatomical study was carried out using eight male mice. There were five WT mice and three HET mice. These mice were 16 weeks of age and bred on a B6N background. A full list of parameters studied in this manuscript is provided as Supplementary Table S3. A number of parameters were significantly decreased or increased compared with WT, and are listed in Supplementary Table S4: the corpus callosum (cc) in section Br −1.34, piriform cortex, right (Pir_R) in section Br +0.98, total brain area (2_TBA) in section Br −1.34, lateral ventricles, right (2_LV_R) in section Br −1.34, dorsal hippocampal commissure (2_dhc) in section Br −1.34, and the arcuate nucleus (2_Arc) in section Br −1.34. Significant parameters were mapped onto the two neuroanatomical sections performed in this study (Figure 6A) and the full spectra of parameters studied in this report are expressed as relative increases or decreases compared with controls in bar graphs (Figure 6A below). Significant neuroanatomical phenotypes at Br. +0.98 mm (right piriform cortex and right secondary somatosensory cortex) and −1.34 mm (total brain area and measurements pertaining to ventricles and the corpus callosum) are shown on representative images of wild-type and mutant animals, together with box plots (Figure 6B,C). The most significant neuroanatomical finding pertained to the size of the corpus callosum that was increased in size by about 20% (*p =* 0.004) (Figure 6B,C). Statistically significant unilateral neuroanatomical phenotypes (oriens layer of the hippocampus, piriform cortex and secondary somatosensory cortex) should be considered with due caution. The raw data are provided in Supplementary Table S5.

## 4. Discussion

Using an analysis of large-scale data from whole-genome SNVs, CNVs, and GWASs studies of SZ patients, we were able to evidence a protein network enriched in products of genes encoding Microtubule-associated proteins (MAPs), actin-interacting proteins, and synaptic proteins. These three groups of proteins interact in the organization of the neuronal cytoskeleton, regulate cellular development, and maintain axons, dendrites, and synapses [30,31,32,33,34,35]. This result fits into a larger theme of cytoskeletal dysregulation in SZ. Such abnormalities in cytoskeletal organization networks have become increasingly implicated in SZ pathogenesis through the evidence of large-scale genomic studies, proteomic analyses, and immunohistochemical assays [18,32,36].

Currently, large-scale genetic studies are dominated by European-descent samples. This restricted population can induce failure to capture the level of diversity that exists globally [37,38]. Due to differential genetic architectures, the universality of genetic findings between populations is generally limited. Therefore, this imbalance poses a limitation in our understanding of the genetic architecture of complex diseases in non-European populations. Here, we analysed three distinct trio cohorts from France, Algeria, and Japan. From these distinct three populations, we observed a similar enrichment in genes that encode products related to G0:0003774~transport, GO:0051015~actin filament binding, and GO:0003729~mRNA binding. One can expect a functional interaction between these three groups of proteins. GO:0003729~mRNA binding proteins are expected to bind mRNAs that are localized either in dendritic spines or axonal boutons and involved in the synaptic plasticity [39,40].

Limitations of our study need to be evaluated. Genomic studies of SZ have revealed a condition that is highly polygenic, with risk conferred by probably thousands of risk alleles, each of small effect [41], pointing to the importance of studying cohorts as large as possible. Our work was based on the analyses of GWAS data that identified 346 genes from the 108 genetic loci found associated with SZ [28] and our 181 SZ trios. To date, GWASs have identified 287 loci associated with SZ [42]. De novo mutations have been validated from 1695 SCZ-affected trios and 1077 published SCZ-affected trios [43]. Inclusions of these data in meta-analysis are expected to refine the analysis of gene networks found in this study.

Neuroimaging reports have found corpus callosum deficits in patients with SZ. Examining neurocircuitry, diffusion-weighted imaging studies have identified an altered structural integrity of white matter in frontal and temporal brain regions and tracts, such as the corpus callosum associated with the illness [44]. One meta-analysis study identified differences in the fractional anisotropy of corpus callosum between patients and controls [45]. Furthermore, multivariate associations among white matter, neurocognition, and social cognition were quantified across individuals with SZ spectrum disorders [46].

Studies from patients-derived neurons also indicate defects in microtubules. Deficits in microtubules organization and stability were evidenced using olfactory neuroepithelial cells from patients with SZ [47,48]. These neurons are expected to express a glutamatergic phenotype [49]. In contrast, no deficits were reported for patient-derived dopaminergic neurons [50]. These results suggest that SZ affects microtubule function, depending on the neurotransmitter phenotype of human neurons.

From these results and the data reported in our manuscript, one can propose that SZ induces defects in microtubule function, impacting long-range projecting glutamatergic neurons such as those implicated in the development of the corpus callosum. This hypothesis should be studied further in the future.

Altogether, our results emphasize the importance of gene networks involved in neuronal cytoskeleton transport and their deregulation linked to de novo mutations in SZ.

## Figures and Tables

**Table 1:** Genes of the network associated with the three first GO categories, namely, Microtubule-Based Process, Synaptic Transmission, and Actin Cytoskeleton.

Microtubule-Based Process (G0:0007017)	Synaptic Transmission (G0:0007268)	Actin Cytoskeleton (GO:0015629)
CLASP1 *(none)*	AKAP9 *(SNV)*	ACTA2 *(SNV)*
CTTNBP2 *(SNV)*	ALDH5A1 *(SNV)*	CAPZA1 *(SNV)*
DCTN3 *(SNV)*	BAIAP3 *(SNV)*	CTNNA2 *(SNV)*
DNAH1 *(SNV)*	CACNA1C *(GWAS)*	DBN1 *(SNV)*
DNAH3 *(SNV)*	CACNB2 *(GWAS)*	DCTN3 *(SNV)*
DNAH9 *(SNV)*	CTNNA2 *(SNV)*	EPB41 *(SNV)*
HAUS6 *(SNV)*	CTTNBP2 *(SNV)*	ESPN *(SNV)*
HAUS8 *(SNV)*	DMD *(SNV)*	KLHL20 *(SNV)*
HTT *(SNV)*	DRD2 *(GWAS)*	MYH11 *(SNV)*
KIF13B *(SNV)*	DVL1 *(SNV)*	MYH9 *(SNV)*
KIF14 *(SNV)*	GABRD *(CNV)*	MYO15A *(GWAS&SNV)*
KIF18A *(SNV)*	GRID2 *(SNV)*	MYO18A *(SNV)*
KIF1A *(cnv&snv)*	GRIN2A *(GWAS&SNV)*	MYO18B *(SNV)*
KIF20B *(SNV)*	GRM3 *(GWAS)*	MYOZ2 *(SNV)*
KIF3A *(SNV)*	HTR2A *(SNV)*	NEB *(SNV)*
KIF6 *(SNV)*	KCNQ5 *(SNV)*	PRKCZ *(CNV)*
KLC1 *(GWAS)*	LPAR3 *(SNV)*	SHROOM1 *(SNV)*
MARK4 *(SNV)*	NRXN1 (SNV)	SLC9A3R1 *(SNV)*
MYH9 *(SNV)*	PTPRF *(GWAS&SNV)*	SORBS2 *(SNV)*
PCNT *(SNV)*	RIMS1 *(SNV)*	SRCIN1 *(SNV)*
PEX1 *(SNV)*	SLC1A1 *(CNV)*	STK38L *(SNV)*
PRKCZ *(CNV)*	SLC5A7 *(SNV)*	TTN *(SNV)*
TNKS *(SNV)*	SLC6A4 *(none)*	UTRN *(SNV)*
TTK *(SNV)*	VDAC1 *(SNV)*	
UBE2C *(SNV)*		

**Table 2:** Gene Ontology classification after DAVID analysis using the NETBAG+ network on SNV and CNV classes.

*N*	*X*	*P*_adj_	GO Identifier	Ontology	GO Term
24	253	0.001	GO:0007017	BP	microtubule-based process
23	269	0.001	GO:0015629	CC	actin cytoskeleton
20	274	0.006	GO:0005874	CC	microtubule
20	244	0.031	GO:0000904	BP	cell morphogenesis involved in differentiation
18	141	<0.001	GO:0008022	MF	protein C-terminus binding
18	246	0.010	GO:0044456	CC	synapse part
16	224	0.025	GO:0005813	CC	centrosome
15	142	0.006	GO:0003774	MF	motor activity
15	205	0.028	GO:0005635	CC	nuclear envelope
14	113	0.001	GO:0044449	CC	contractile fiber part
14	121	0.002	GO:0043292	CC	contractile fiber
14	147	0.006	GO:0005819	CC	spindle
13	135	0.008	GO:0045211	CC	postsynaptic membrane
13	140	0.041	GO:0005516	MF	calmodulin binding
12	98	0.003	GO:0030017	CC	sarcomere
12	111	0.006	GO:0030016	CC	myofibril
10	71	0.005	GO:0014069	CC	postsynaptic density
10	77	0.023	GO:0003777	MF	microtubule motor activity

**Table 3:** Distribution of the de novo mutations per cohorts.

	Algerian Cohort	French Cohort	Japanese Cohort	Total
**Number Of Families**	75	45	61	181
**Number Of Mutations**	35	21	32	88
**Number Of SZ Patients With Mutation(s)**	30	17	25	72
**Number Of Chromosomes With Mutation(s)**	18	14	16	22

**Table 4:** Distribution of the de novo mutations per SZ patient.

	Algerian Cohort	French Cohort	Japanese Cohort
**% SZ Patients With 1 Mutation**	36	31	31
**% SZ Patients With 2 Or More Mutations**	4	7	10
**% SZ Patients With Mutation(s)**	40	38	41

**Table 5:** Distribution of the de novo mutations per chromosome.

	Algerian Cohort	French Cohort	Japanese Cohort
**% Chromosomes With 1 Mutation**	52	39	39
**% Chromosomes With 2 Mutations**	4	17	4
**% Chromosomes With 3 Or More Mutations**	22	4	26
**% Chromosomes With Mutation(s)**	78	61	70

**Table 6:** Distribution of the de novo mutations per PP2 ranking.

	Mutations Probably Damaging	Mutations Possibly Damaging	Mutations Benign	Total
**Algerian Cohort**	19	6	10	35
**French Cohort**	17	0	4	21
**Japanese Cohort**	12	13	7	32
**Total**	48	19	21	88

**Table 7:** List of de novo mutations validated by Sanger sequencing.

Cohort	Gene	Chromosome	Position	Father	Mother	Patient	PPH2 Score	PPH2 Prediction
Algerian	HISPPD1	5	chr5:102489584	G/G	G/G	G/A	1	probably damaging
Algerian	BTRC	10	chr10:103310478	G/G	G/G	G/A	1	probably damaging
Algerian	SKP2	5	chr5:36181963	G/G	G/G	G/A	1	probably damaging
Algerian	PUM1	1	chr1:31414074	G/G	G/G	A/G	1	probably damaging
Algerian	ZNF618	9	chr9:116811165	G/G	G/G	G/A	1	probably damaging
Algerian	PCDH19	X	chrX:99597014	C/C	C/C	T/T	1	probably damaging
Algerian	ACOX1	17	chr17:73944381	T/T	T/T	A/T	1	probably damaging
Algerian	ATP13A2	1	chr1:17318848	G/G	G/G	A/G	1	probably damaging
French	QTRT1	19	chr19:10822884	G/G	G/G	G/T	1	probably damaging
French	WDR53	3	chr3:196287910	G/G	G/G	A/G	1	probably damaging
French	MC3R	20	chr20:54824818	C/C	C/C	C/T	1	probably damaging
French	CES4	16	chr16:55806311	G/G	T/T	G/G	1	probably damaging
French	CSPP1	8	chr8:68070693	G/G	G/G	G/C	1	probably damaging
French	LEO1	15	chr15:52245429	C/C	C/C	G/C	1	probably damaging
French	CORO1C	12	chr12:109041222	C/C	C/C	C/T	1	probably damaging
Japanese	ABCC2	chr10	chr10:101611278	G/G	G/G	G/A	1	probably damaging
Algerian	LPHN1	19	chr19:14272190	G/G	G/G	G/A	0.999	probably damaging
French	KRT77	12	chr12:53086645	G/G	G/A	A/A	0.999	probably damaging
French	HLA-H	6	chr6:29855818	T/T	G/G	G/G	0.999	probably damaging
French	VEPH1	3	chr3:157177981	C/C	C/C	T/C	0.999	probably damaging
Japanese	ITGAM	chr16	chr16:31332562	C/C	C/C	C/T	0.999	probably damaging
Japanese	IL17B	chr5	chr5:148754050	G/G	G/G	G/A	0.999	probably damaging
Algerian	LPCAT1	5	chr5:1494826	G/G	G/G	G/A	0.997	probably damaging
Japanese	SECISBP2	chr9	chr9:91964831	C/C	C/C	C/T	0.997	probably damaging
French	NIN	14	chr14:51259598	G/G	G/G	T/G	0.996	probably damaging
Japanese	PRRG3	chrX	chrX:150869475	A/A	A/A	A/G	0.994	probably damaging
Algerian	GLI1	12	chr12:57864819	T/T	T/T	T/A	0.993	probably damaging
Japanese	DHRS9	chr2	chr2:169940028	G/G	G/G	G/A	0.993	probably damaging
French	SCUBE3	6	chr6:35207648	C/C	C/C	G/C	0.988	probably damaging
Japanese	GPR83	chr11	chr11:94113926	G/G	G/G	G/A	0.987	probably damaging
French	STK38L	12	chr12:27462071	A/A	A/A	G/A	0.985	probably damaging
Algerian	SRRM1	1	chr1:24977932	G/G	G/G	A/G	0.983	probably damaging
Algerian	POTEA	8	chr8:43157139	G/G	G/G	A/G	0.982	probably damaging
Japanese	MARK4	chr19	chr19:45790886	G/G	G/G	G/T	0.98	probably damaging
Algerian	OR4C11	11	chr11:55371021	T/T	G/G	T/T	0.977	probably damaging
French	IPO13	1	chr1:44415659	C/C	C/C	T/C	0.977	probably damaging
Algerian	MYH11	16	chr16:15820797	T/T	T/T	T/G	0.976	probably damaging
French	DNAH10	12	chr12:124359929	A/A	A/A	G/A	0.976	probably damaging
Japanese	HIPK2	chr7	chr7:139268761	C/C	C/C	C/T	0.972	probably damaging
Algerian	C11orf36	11	chr11:3243412	A/A	C/C	A/A	0.97	probably damaging
French	SPEN	1	chr1:16262042	C/C	C/C	T/C	0.969	probably damaging
Algerian	BTNL3	5	chr5:180420151	C/C	T/T	T/T	0.963	probably damaging
French	OR4S2	11	chr11:55418693	C/C	T/T	T/T	0.96	probably damaging
Algerian	OR4P4	11	chr11:55406157	A/A	G/G	A/A	0.959	probably damaging
Japanese	RBM14	chr11	chr11:66392695	G/G	G/G	G/A	0.959	probably damaging
Algerian	MYO1B	2	chr2:192228467	T/T	T/T	C/T	0.949	probably damaging
Japanese	GABRR1	chr6	chr6:89910913	T/T	T/T	T/C	0.948	probably damaging
Japanese	INTS3	chr1	chr1:153745469	G/G	G/G	G/A	0.947	probably damaging
Algerian	TTN	2	chr2:179585273	C/C	C/C	C/A	0.929	possibly damaging
Algerian	ADAMTS2	5	chr5:178634558	C/C	C/C	C/T	0.921	possibly damaging
Japanese	OR9G9	chr11	chr11:56468440	G/G	G/G	G/T	0.899	possibly damaging
Japanese	PRIM2	chr6	chr6:57512510	G/G	G/G	T/G	0.892	possibly damaging
Japanese	DNAH6	chr2	chr2:84848382	C/C	C/C	C/A	0.864	possibly damaging
Japanese	TCP11L2	chr12	chr12:106740187	A/A	A/A	A/G	0.823	possibly damaging
Japanese	KIAA1009	chr6	chr6:84910572	T/T	T/T	T/G	0.801	possibly damaging
Japanese	LAD1	chr1	chr1:201356296	C/C	C/C	C/T	0.783	possibly damaging
Japanese	RTTN	chr18	chr18:67691972	G/G	G/G	G/A	0.763	possibly damaging
Algerian	RYR3	15	chr15:33955901	G/G	G/G	G/A	0.718	possibly damaging
Japanese	CAST	chr5	chr5:96097979	A/A	A/A	A/T	0.708	possibly damaging
Japanese	DAB2	chr5	chr5:39392558	G/G	G/G	G/A	0.666	possibly damaging
Algerian	UHRF1	19	chr19:4941574	G/G	G/G	A/G	0.651	possibly damaging
Algerian	PCDHB9	5	chr5:140568167	A/A	T/T	T/T	0.645	possibly damaging
Japanese	SNED1	chr2	chr2:241976318	G/G	G/G	G/A	0.623	possibly damaging
Japanese	NACC1	chr19	chr19:13249035	G/G	G/G	G/A	0.569	possibly damaging
Japanese	FOXI1	chr5	chr5:169535610	G/G	G/G	G/A	0.556	possibly damaging
Algerian	NFIC	19	chr19:3381984	C/C	C/C	T/C	0.519	possibly damaging
Japanese	TRDN	chr6	chr6:123851705	G/G	G/G	G/A	0.478	possibly damaging
Japanese	CLSPN	chr1	chr1:36230927	C/C	C/C	C/A	0.414	benign
Japanese	FAM178A	chr10	chr10:102672962	C/C	C/C	C/T	0.398	benign
Algerian	ZIC1	3	chr3:147128576	T/T	T/T	C/T	0.391	benign
Algerian	ZNF548	19	chr19:57910901	A/A	A/A	G/A	0.33	benign
Japanese	DCAF4	chr14	chr14:73406575	C/C	C/C	C/T	0.205	benign
French	SKP2	5	chr5:36182122	A/A	A/A	C/A	0.181	benign
Japanese	CCAR1	chr10	chr10:70508947	G/G	G/G	G/A	0.162	benign
Japanese	VPS25	chr17	chr17:40931073	G/G	G/G	G/A	0.144	benign
Japanese	SPRED2	chr2	chr2:65559158	T/T	T/T	T/C	0.113	benign
Japanese	SMC1B	chr22	chr22:45767391	C/C	C/C	C/T	0.104	benign
French	RBM20	10	chr10:112572199	G/G	G/G	G/T	0.07	benign
Algerian	SPRY4	5	chr5:141694466	C/C	C/C	T/C	0.029	benign
French	ARHGAP21	10	chr10:24908686	C/C	T/T	C/C	0.013	benign
Algerian	VASN	16	chr16:4430931	G/G	G/G	A/G	0.004	benign
French	UGGT2	13	chr13:96665620	C/C	C/C	T/C	0.004	benign
Algerian	BTBD7	14	chr14:93709344	C/C	C/C	A/C	0.002	benign
Algerian	PCDHB9	5	chr5:140567445	A/A	A/G	G/G	0.001	benign
Algerian	SYNE1	6	chr6:152673248	T/T	T/T	T/C	0.001	benign
Algerian	SCNN1G	16	chr16:23200844	G/G	G/G	A/G	0.001	benign
Algerian	DSCR3	21	chr21:38612937	C/C	C/C	G/C	0	benign
Algerian	TNFRSF6B	20	chr20:62329635	G/G	G/G	A/G	0	benign

## Data Availability

Coro1c^tm1a^ are available through Infrafrontier (https://www.infrafrontier.eu/).

## Supplementary Materials

The following supporting information can be downloaded at: https://www.mdpi.com/article/10.3390/life14020244/s1, Figure S1: Complete network from NETBAG+. Tables S1–S5.
