# Supplementary material for: De Novo Variants Found in Three Distinct Schizophrenia Populations Hit a Common Core Gene Network Related to Microtubule and Actin Cytoskeleton Gene Ontology Classes
Source: Life (Basel). 2024 Feb 9;14(2):244. doi: 10.3390/life14020244 (PMC10890674; doi:10.3390/life14020244)
Supplement: Supplementary file 1 [file life-14-00244-s001.zip › Supplementary Figure 1.docx]

**Supplementary Figure 1. Complete network from NETBAG+.** The size of circles depends on the number of connections. The color code for the different gene ontology classes is: yellow for synapse related, pink for actin cytoskeleton, blue for Microtubule based process and grey blue for Microtubule based process & actin cytoskeleton.
