# Supplementary figures and images for: De Novo Variants Found in Three Distinct Schizophrenia Populations Hit a Common Core Gene Network Related to Microtubule and Actin Cytoskeleton Gene Ontology Classes
Source: Life (Basel). 2024 Feb 9;14(2):244. doi: 10.3390/life14020244 (PMC10890674; doi:10.3390/life14020244)

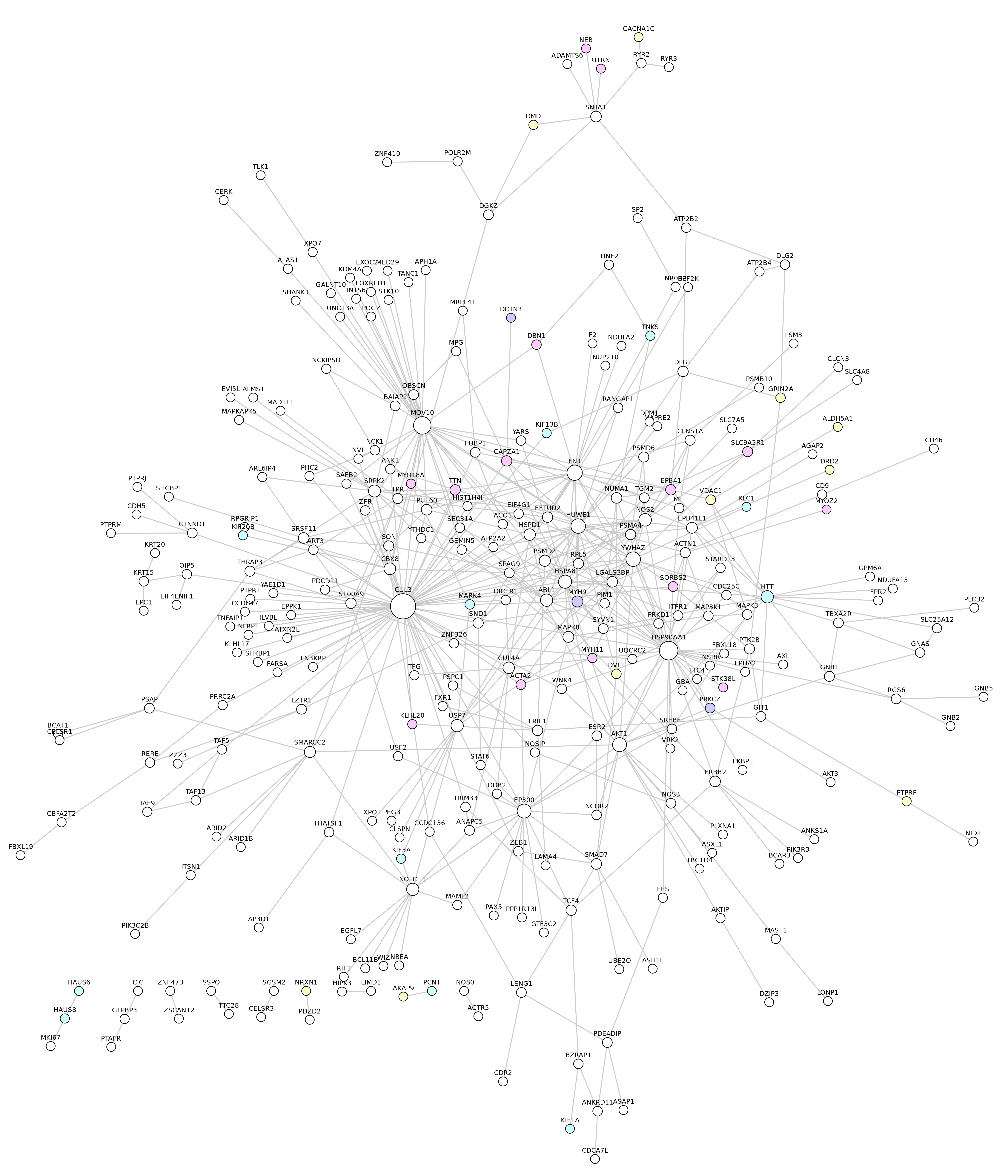

Supplement: Supplementary file 1 [file life-14-00244-s001.zip › Fig S1 complete_network.png]
